# Early Circulation of SARS-CoV-2, Congo, 2020

**DOI:** 10.3201/eid2804.212476

**Published:** 2022-04

**Authors:** Novy Charel Bobouaka Bonguili, Matthieu Fritz, Leadisaelle Hosanna Lenguiya, Pembe Issamou Mayengue, Félix Koukouikila-Koussounda, Louis Régis Dossou-Yovo, Cynthia Nkoua Badzi, Eric M. Leroy, Fabien R. Niama

**Affiliations:** Laboratoire National de Santé Publique, Brazzaville, Republic of Congo (N.C. Bobouaka Bonguili, L.H. Lenguiya, P. Issamou Mayengue, F. Koukouikila-Koussounda, L.R. Dossou-Yovo, C. Nkoua Badzi, F.R. Niama);; Université Marien Ngouabi, Brazzaville (N.C. Bobouaka Bonguili, L.H. Lenguiya, P. Issamou Mayengue, F. Koukouikila-Koussounda, L.R. Dossou-Yovo, F.R. Niama);; Université de Montpellier, Montpellier, France (M. Fritz, E.M. Leroy);; Institut de Recherche pour le Développement, Unité Mixte de Recherche MIVEGEC, Montpellier (M. Fritz, E.M. Leroy)

**Keywords:** COVID-19, 2019 novel coronavirus disease, coronavirus disease, severe acute respiratory syndrome coronavirus 2, SARS-CoV-2, viruses, respiratory infections, zoonoses, Central Africa, Republic of Congo, Luminex, serology, retrospective study, microsphere immunoassay

## Abstract

To determine when severe acute respiratory syndrome coronavirus 2 arrived in Congo, we retrospectively antibody tested 937 blood samples collected during September 2019–February 2020. Seropositivity significantly increased from 1% in December 2019 to 5.3% in February 2020, before the first officially reported case in March 2020, suggesting unexpected early virus circulation.

After coronavirus disease (COVID-19) was reported in China in December 2019, severe acute respiratory syndrome coronavirus 2 (SARS-CoV-2) rapidly spread around the world; most countries officially reported their first cases within the first 3 months of 2020. However, reports from China show a possible earlier first case on November 17, 2019, detected retrospectively in Hubei Province (*1*). Furthermore, phylogenetic analysis places the date of emergence as sometime during October–December 2019 (*2*). These data suggest possible virus spread outside China before the first officially reported case in December 2019. Indeed, several retrospective studies that analyzed stored respiratory samples and wastewater for RNA detection, as well as serologic studies, suggest that SARS-CoV-2 may have been circulating in France, Spain, and Italy (*3*–*7*) before December 2019, months before the first official cases were reported. 

In central Africa, the first cases were officially reported during March 6–April 6, 2020; in Congo, the first case was reported on March 14, 2020. However, a serologic study in Kenya suggested that the virus was present in January 2020, two months before the first official case was reported (*8*). Similar retrospective studies have not been conducted in Central Africa, meaning that the time of SARS-CoV-2 introduction in this region remains unknown.

To provide a more accurate date for the arrival of SARS-CoV-2 in Congo, we retrospectively examined serum samples collected from persons with HIV (PWH) as a part of the national HIV program. These samples were collected during July 2019–February 2020 in Brazzaville and Pointe-Noire, the 2 biggest cities in Congo (Appendix). The study was conducted with approval of the Comité Technique de la Riposte à la Maladie à Coronavirus COVID-19, of which F.R.N. is president of the commission laboratory and research, and the Programme National de Lutte Contre le SIDA, led by the National Public Health laboratory of Congo, of which F.R.N. is director.

We tested 1,212 plasma samples for SARS-CoV-2 IgG by using a microsphere immunoassay with beads coupled with receptor-binding domain antigen. We used 275 samples collected during July–August 2019 as negative controls and to establish the seropositivity cutoff value of our test (Appendix). The remaining 937 samples were collected September 2019–February 2020. Overall, 28/937 (3.0%) samples were positive: 22/655 (3.3%) from women, 5/241 (2.1%) from men, and 1/41 (2.4%) from a patient for whom sex was not reported. SARS-CoV-2 seropositivity rate was 1.7% (10/563) in Brazzaville and 4.8% (18/374) in Pointe-Noire. However, the Pointe-Noire samples were all collected in 2020 and compared with those from Brazzaville from the same period (5.4%; 6/110) did not differ significantly (p = 0.8). Although seropositivity was very low from September through November, seropositivity subsequently increased linearly, reaching 5.3% by February 2020 (Figure). Furthermore, seropositivity was significantly higher in January–February 2020 (p = 0.0002) than in the preceding 4 months of 2019 (Table). We also observed a significant increase between samples collected in Brazzaville in 2019 and those collected in Brazzaville in 2020 (p = 0.0052).

**Figure:**
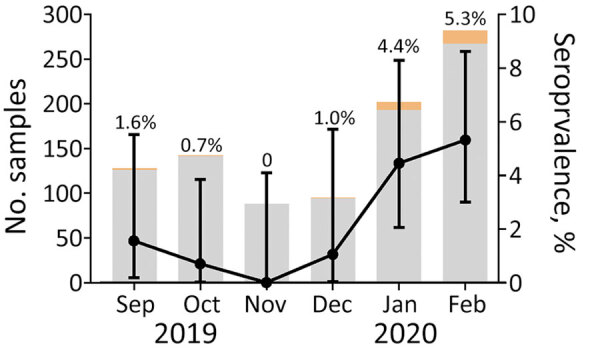
Number of plasma samples tested each month for severe acute respiratory syndrome coronavirus antibodies by using a microsphere immunoassay with beads coupled with receptor-binding domain antigen, Congo, September 2019–February 2020. Gray shading indicates the number of seronegative samples; orange, seropositive samples. Seropositivity is represented by black dots; error bars indicate 95% binomial CIs.

**Table:** Seropositivity of SARS-CoV-2 IgG among persons with HIV, Congo, July 2019–February 2020*

Sample	No. tested/no positive (% positive)	p value
Sex		
F	655/22 (3.3)	0.38
M	241/5 (2.1)	
Unknown	41/1 (2.4)	NA
Location		0.0052 for Brazzaville 2020 vs. 2019; 0.8 for Brazzaville 2020 vs. Pointe-Noire 2020
Brazzaville 2019	453/4 (0.8)	
Brazzaville 2020	110/6 (5.4)	
Pointe-Noire 2020	374/18 (4.8)	
Date		
2019 September–December	453/4 (0.9)	0.0002
2020 January–February	484/24 (4.9)	
Total	937/28 (3.0)	NA

*NA, not applicable; SARS-CoV-2, severe acute respiratory syndrome coronavirus 2.

Our results suggest increased SARS-CoV-2 circulation during January–February 2020 in Congo, indicating that the virus arrived in the country in December 2019. Our findings align with those of a serologic study of an asymptomatic general population in Congo, conducted in April 2020, which found 1.7% seropositivity for IgG and 2.5% for IgM (*9*). The higher seropositivity found before April in our study may result from the higher sensitivity of the microsphere immunoassay assay compared with that of rapid tests (*9*). Moreover, the PWH in our study may be more exposed to the virus than the randomized general population tested by Batchi-Bouyou et al. because PWH must regularly visit healthcare centers as part of their treatment. A recent study of participants with and without HIV tested during January–March 2020 in Kenya reported 3%–4% seropositivity, which did not differ between these populations (*8*). Early circulation of SARS-CoV-2 has also been found in France, Spain, and Italy; seropositivity estimates in France increased from 1.3% in November 2019 to 6.7% in February 2020 (*6*).

There is some concern that seropositive samples may reflect possible cross-reactions with other coronaviruses that infect humans (human coronaviruses NL63, 229E, OC43, and HKU1 and Middle East respiratory system coronavirus) (*10*). Although cross-reaction may explain the very low SARS-CoV-2 seropositivity in September–October 2019, the significant increase in seropositivity from the end of 2019 to the beginning of 2020 argues in favor of actual detection of antibodies directed against SARS-CoV-2. The early introduction of SARS-CoV-2 in Congo, and more generally in Africa, probably results from the intense trade activities that link Africa to China, leading to frequent exchange of persons between these countries.

Determining early circulation patterns of SARS-CoV-2 in Africa or other countries requires retrospective testing of as many samples as possible from existing national sample repositories. Such studies will help enrich knowledge of the propagation of pathogens in the context of globalization of human and material exchange. To better evaluate the epidemiology of future pandemics, international organizations should help reinforce and develop repositories in low- and middle-income countries.

Appendix Supplemental methods for study of early circulation of SARS-CoV-2, Congo, 2020.
