# Supplementary material for: Early Circulation of SARS-CoV-2, Congo, 2020
Source: Emerg Infect Dis. 2022 Apr;28(4):878–80. doi: 10.3201/eid2804.212476 (PMC8962888; doi:10.3201/eid2804.212476)
Supplement: Appendix — Supplemental methods for study of early circulation of SARS-CoV-2, Congo, 2020. [file 21-2476-Techapp-s1.pdf]

Article DOI: https://doi.org/10.3201/eid2804.212476

Serologic Evidence for SARS-CoV-2 Circulation in Early 2020, Congo

Appendix

Material and methods

Origin of sample

Plasma samples were gathered from the PWH library of the National Public Health Laboratory in Brazzaville. The library contains samples collected in the various outpatient treatment centers in the Congo and samples from PWH received at the LNSP from July 2019 to February 2020. Only patients receiving ARV treatment and from Brazzaville and Pointe-Noire were choose for this study. No other inclusion criteria has been applied for this study. The average age of the patients was 46 years (range: 17 to 80), 72% were women, 24.5% were men, and for 3.5% the information on gender was unavailable.

Microsphere immunoassay

The plasma samples were tested using a Microsphere immunoassay (MIA). 10µg of Spike receptor binding domain (RBD) recombinant SARS-CoV-2 antigen (The Native Antigen Company) was used to capture specific plasma antibodies. MagPlex microsphere (Luminex Corp) was coupled to the viral antigen using the amine coupling kit (Bio-Rad Laboratories) according to manufacturers' instructions. The MIA procedure was performed by incubating the plasma samples (50 µl), diluted 1:400 in assay buffer (PBS-1% BSA-0.05% Tween 20), with the antigen-coated beads (1250 beads) protected from the light on an orbital shaker at 700 rpm for 30 min. After washing, 50 µl of biotinylated conjugated Fc Fragment goat anti-Human IgG (Jackson ImmunoResearch) at 4 µg/ml each in assay buffer were transferred to each well and incubated on an orbital shaker for 30 min at 700 rpm in the dark. After washing, the beads were incubated for 10min at 700 rpm in the dark with 50 µl of Streptavidin-R-Phycoerythrin (Life technologies) and diluted to 4 µg/mL in assay buffer. After washing, beads were resuspended in 125 µl of assay

buffer. Measurements were performed using a Magpix instrument (Luminex), with at least 50 events were read. Binding events were displayed as median fluorescence intensities (MFI).

Calculation of the cutoff, specificity and sensitivity

To take into account antigen specificity, seropositivity cutoff values was set at three standard deviations above the mean MFI of 275 plasma samples available from this cohort farthest from the beginning of the epidemic, i.e., in July and August 2019. Based on this population, MIA specificity was set at 97,5%. The sensitivity of our test was set up using samples (12) from COVID-19 PCR+ confirmed non-HIV participant sampled within 2 weeks after the PCR test. Based on this population, MIA sensitivity was set at 100%.

Statistical analyses

Fisher exact test and binomial confidence intervals was calculated using R software (*1*).

Reference

1. R Development Core Team. R: A language and environment for statistical computing. Vienna, Austria: R Foundation for Statistical Computing; 2018.
